# Parliament and Publications

**Published:** 1911-05-13

**Authors:** 


### Parliament and Publications.

### VACCINATION SHIELDS.

Sir Harry Samuel mentioned in the House of Commons that public vaccinators are using shields or vaccination-pads made in Germany, and he asked whether steps would be taken for them to obtain an equally good article manufactured in this country. Mr. Burns replied that no special type of covering was prescribed, and he had no information as to whether any of these coverings were made in Germany.

### ARMY MEDICAL CORPS: MANUAL OF INSTRUCTIONS.

Replying to a question asked by Mr. Cautley, Colonel Seely said the manual of instructions for the Army Medical Corps training was out of print, but a few copies were in stock at the War Office for special issue to officers requiring them for examination purposes. The new edition will, it is hoped, be ready for press in a few weeks' time.

### FEES OF PUBLIC VACCINATORS.

The President of the Local Government Board said he was aware that all the public vaccinators at Bermondsey receive a fee of 6s. for each vaccination they successfully perform. The minimum fee under the order of the Local Government Board is 2s. 6d., but the actual fee to be paid is ordinarily a matter of arrangement between the guardians and the public vaccinator. Vaccinations when done by medical students are done under the direct personal supervision of the public vaccinator, and in exactly the same way as other vaccinations are done.

